# ADBrainNet: a deep neural network for Autism Spectrum Disorder (ASD) and Attention Deficit and Hyperactivity Disorder (ADHD) classification using resting-state fMRI images based on explainable artificial intelligence

**DOI:** 10.1007/s11517-026-03530-2

**Published:** 2026-03-05

**Authors:** Xinyao Yi, Jian Huang, Babatunde Akinwunmi, Wai-kit Ming

**Affiliations:** 1 Department of Infectious Diseases and Public Health, Jockey Club College of Veterinary Medicine and Life Sciences, City University of Hong Kong, Hong Kong SAR, China; 2 Institute for Human Development and Potential (IHDP), Agency for Science, Technology and Research (A*STAR), Singapore, Republic of Singapore; 3 Bioinformatics Institute (BII), Agency for Science, Technology and Research (A*STAR), Singapore, Republic of Singapore; 4 Human Potential Translational Research Programme, Yong Loo Lin School of Medicine, National University of Singapore, Singapore, Republic of Singapore; 5 Department of Obstetrics and Gynecology, Queens Hospital Center, New York, US; 6 Institute of Global Governance and Innovation for a Shared Future, City University of Hong Kong, Hong Kong SAR, China; 7 City University of Hong Kong Shenzhen Research Institute, Shenzhen, China

**Keywords:** Deep neural network, Resting-state fMRI, ASD, ADHD, Explainable artificial intelligence

## Abstract

Autism Spectrum Disorder (ASD) and Attention Deficit and Hyperactivity Disorder (ADHD) are two psychiatric disorders frequently encountered in children. ADHD is further categorized into three subtypes. The diagnostic processes for these conditions are complex and often prone to misclassification. We proposed a lightweight deep neural network, ADBrainNet, to differentiate ASD, ADHD combined, ADHD hyperactive/impulsive, ADHD inattentive and neurotypical individuals. Our methodology was benchmarked against prevalent ImageNet transfer learning methods, including AlexNet, MobileNet, ResNet18, and Xception, for training on resting-state fMRI images sourced from ABIDE and ADHD-200 datasets. ADBrainNet achieved superior performance on the independent external testing set through five-fold cross-validation, with a mean (± standard deviation) accuracy, precision, recall, and F1 score of 61.87% (± 5.59%), 65.72% (± 6.98%), 61.87% (± 5.59%), and 62.50% (± 5.78%), respectively. Furthermore, the explainable artificial intelligence algorithm LIME was employed to explore the most significant features during ADBrainNet’s decision process. Our model provides an interpretable computational framework for neuroimaging-based classification between ASD and ADHD subtypes. This approach may inform future research and, upon further validation and comparison with clinician performance, could potentially aid in patient assessment, stratification, and management of psychiatric disorders.

**Graphical abstract:**

**Figure:**
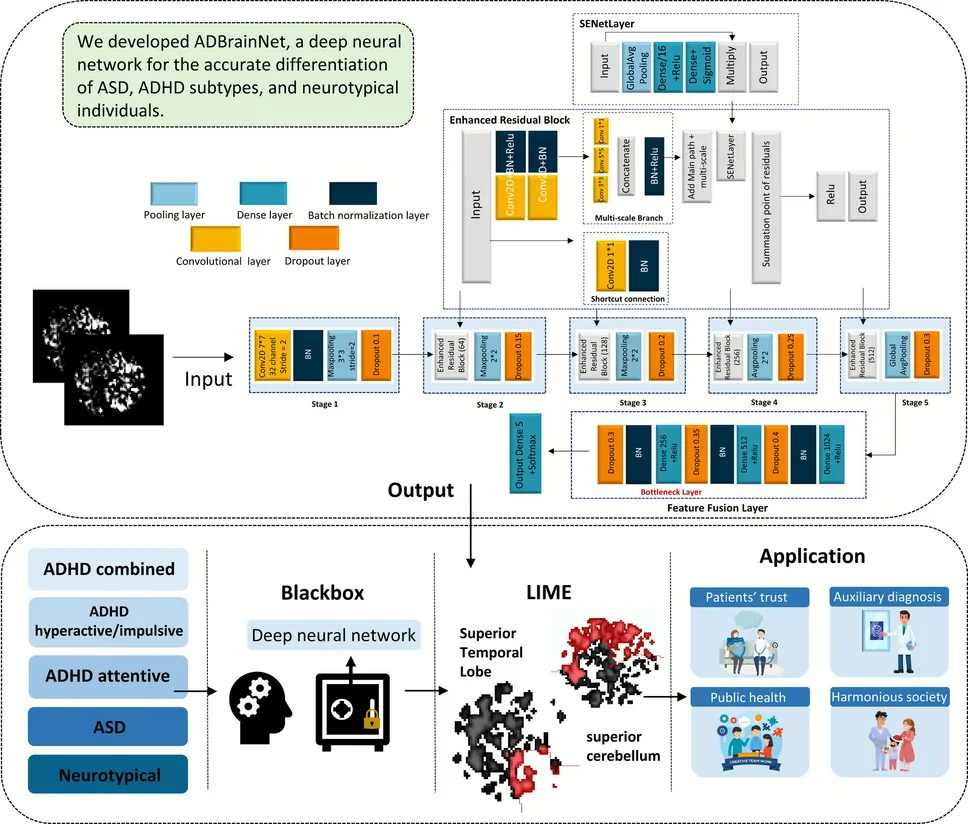

**Supplementary Information:**

The online version contains supplementary material available at https://doi.org/10.1007/s11517-026-03530-2.

## Introduction

ASD, Autism Spectrum Disorder, which is a representative disorder of pervasive developmental disorders (PDD). Its characteristics encompass challenges in social activities and communication skills, coupled with nontraditional patterns of activities and behaviors. About one in every 100 children has ASD according to World Health Organization [1]. ADHD, Attention Deficit Hyperactivity Disorder, manifests as a persistent tendency of inattention and/or hyperactivity-impulsivity, significantly affecting academic, occupational, and social performance. A global meta-analysis of 175 studies across various regions examined the prevalence of ADHD in individuals aged 18 and below, reporting a combined estimate of 7.20% by Thomas et al. [2]. Due to the prolonged and intricate diagnostic process, MRI has emerged as a highly beneficial asset for the auxiliary diagnosis of these diseases.

**Fig. 1:**
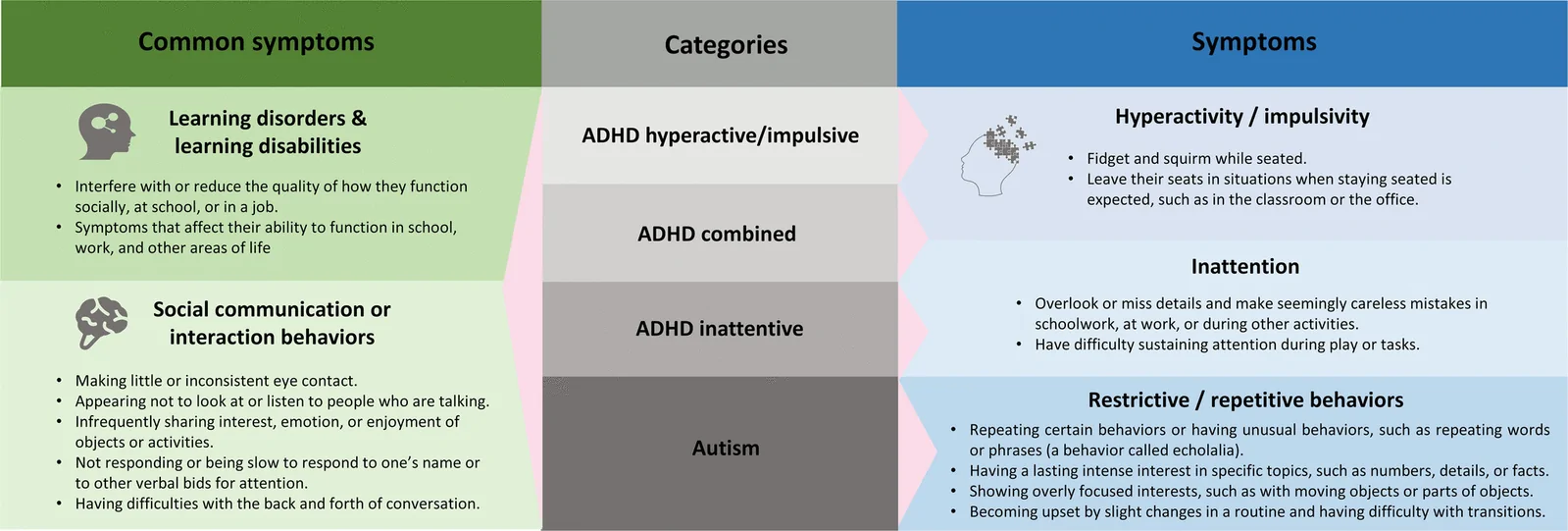
Symptom profiles: similarities and distinctions between ASD and ADHD (combined, hyperactive/impulsive, and inattentive presentations)

Evaluation in behaviors reveal considerable similarities between ASD and ADHD with its subtypes, which frequent co-occurrence noted in the literature [3], showing difficulty and complexity in diagnosis these two diseases in clinical scenarios (Fig. 1). Furthermore, erroneous treatments may exacerbate the conditions in some cases. Given the distinct treatment approaches and underlying mechanisms of these two disorders, solely relying on questionnaire adjustments, family interviews conducted by physicians, and behavioral observations by their family members proves to be time-consuming and inefficient. However, emerging research indicates subtle distinctions in MRI and related imaging techniques between these two neurological disorders. In the grayscale images of resting-state fMRI, varying levels of gray intensity signify distinct degrees of cerebral activity. The fMRI technique operates by discerning alterations in blood circulation associated with neural activity [4, 5]. Therefore, identifying the specific imaging patterns that differentiate these mental health conditions represents a promising research direction warranting further investigation [6].

In contemporary times, artificial intelligence, particularly deep neural networks, have risen as a potent instrument for detecting mental disorders through MRI image analysis in clinical applications [7]. However, most of the existing research focuses on single disease and lacks interpretability and portability. The classification of multiple closely related diseases is of paramount importance for further investigation. By delineating the distinctions between these disorders, neural networks can potentially provide clinicians with invaluable insights into disease mechanisms, symptomatology, and treatment response variability. Moreover, interpretability plays a pivotal role in elucidating AI-driven decisions for clinicians. By comprehending the inner workings of AI models in analyzing medical images for diagnosis, the healthcare providers can foster greater trust in the technology. LIME is an interpretable artificial intelligence algorithm designed to illuminate the decision-making process of black-box models [8]. Its development is geared towards mitigating the interpretability challenges posed by intricate models such as deep learning framework, thereby empowering users to discern the rationale behind a model’s predictions on specific instances. This approach aligns with methodologies observed in numerous studies focusing on image recognition and analysis [9–12]. Furthermore, this transparency holds paramount importance in the integration of AI into clinical settings, where diagnostic decisions necessitate clarity and justification [13]. When AI models furnish explanations for their diagnoses, they serve as invaluable decision-support aids for physicians. Furthermore, lightweight neural networks in clinical diagnosis also lies in their simplicity and efficiency. They can be deployed on various devices to achieve rapid analysis of patient data, which assists physicians to make accurate and efficient treatment decisions.

## Related work

We aim to find the distinct patterns between ASD and ADHD which can be used for improving diagnosis accuracy. Mozhdefarahbakhsh et al. developed a CNN model for binary classification to identify the differences between these two diseases. They achieved 0.92 accuracy on the validation set reflects a commendable level of model fitting [14]. Nevertheless, the testing dataset holds significance as it facilitates a more comprehensive performance evaluation, particularly concerning the generalization to unseen data. Multi-task learning paradigm was also proposed by Huang et al. for short-term diagnosis between ASD and ADHD, 0.687 ± 0.005 mean binary classification accuracy was achieved for ASD datasets and for ADHD dataset, the mean accuracy was 0.650 ± 0.014 [15]. These results demonstrated the robust performance of their models in handling these two complex disorders. However, they actually omitted explanations for clinical practitioners and external testing sets could be used to verify the accuracy and generalization of their models. None of them have considered classifying ASD along with the ADHD subtypes: the combined, inattentive and hyperactive/impulsive subtypes.

Some studies have also focused solely on single disease. Eslami et al. introduced the Auto-ASD-Network model, designed to classify neurotypical and ASD individuals. They used exclusively fMRI data and achieved a highest accuracy of 80.00% on the testing sets [16]. In order to execute the identification of ASD individuals, Subah et al. suggested a deep neural network model which achieved 88.00% mean accuracy [17]. Shao et al. combined graph convolutional network and deep feature selection methods to distinguish individuals with ASD. On the testing set, they achieved an accuracy of 79.50% [18]. Feng et al. unveiled an advanced convolutional neural network to identify ASD. They achieved an accuracy of 99.39%, the precision, recall, and F1 score are 99.85%, 98.80% and 99.32% separately [19]. Heinsfeld et al. proposed a deep neural network and a machine learning method to identify ASD and achieved 70.00% accuracy [20]. Nogay et al. conducted two quadruple and one octal classification employing a deep learning approach. The three models achieved 80.94%, 85.42%, and 67.94% accuracies respectively [21]. Elakkiya et al. also introduced two state-of-the-art models, AutiNet and MinAutiNet, for classifying ASD subjects, achieving accuracies of 77.78% and 88.89% respectively [22].

The deep learning method was modified by Kuang and He to predict the subjects between ADHD and neurotypical individuals as control, combined, inattentive or hyperactive. They achieved the best accuracy as 80.88% [23]. A time-series 3D frames was proposed by Mao et al. to classify ADHD and neurotypical individuals. They achieved an 71.30% accuracy and an 0.80 AUC [24]. An end-to-end deep learning framework deepfMRI to identify ADHD was suggested by Riaz et al. and it achieved an accuracy of 73.10%, a specificity of 91.60%, and a sensitivity of 65.50% [25]. A pre-trained convolutional neural network of ResNet-50 type was employed to classify ADHD and neurotypical individuals by Uyulan et al.. The results showed an accuracy of 93.45%, using the self-collected data with ten-fold cross-validation [26]. A deep learning-based method was utilized to classify the TDC and ADHD groups by Firouzi et al.. Their results showed an accuracy of 76.10% [27]. Dey et al. also introduced a fresh methodology for ADHD diagnosis based on resting-state fMRI images, employing a combination of Capsule Network and LSTM Network. KKI dataset achieved an accuracy of 80.00% and Peking-I dataset attained 73.33% accuracy [28].

## Methods

### Data source

All datasets were obtained from NITRC program (http://www.nitrc.org). The NeuroImaging Tools and Resources Collaboratory is a web-based resource recognized by the United States Department of Health and Human Services for its excellence. It provides comprehensive information, on a continuously expanding range of neuroinformatic software and data.

The ADHD subjects included were obtained from ADHD-200 dataset which aims to expedite understanding of ADHD’s neural underpinnings through open data-sharing and discovery-driven research. 973 subjects with resting-state fMRI and anatomical images were obtained from 8 locations. 585 subjects were sourced from neurotypical individuals, while 362 were from ADHD individuals, and 26 subjects’ statuses yet to be determined. The ADHD subjects were also divided to three subtypes, 212 ADHD combined subjects, 137 ADHD inattentive subjects and 13 ADHD hyperactive/impulsive subjects.

ASD subjects included were downloaded from ABIDE dataset. ABIDE encompassed 17 global locations, collaborating to share resting-state fMRI that have been previously collected, also anatomical and phenotypic datasets. This initiative resulted in 1,112 dataset subjects, with 539 originating from individuals diagnosed with ASD. The remaining 573 subjects are neurotypical individuals. The dataset compilation is contributed by various esteemed medical and research institutions, ensuring that the data labels are meticulously annotated by medical professionals. Demographic distribution of these two datasets between these five classes is shown in Table S1.

Due to the different sources and sites of the ABIDE and ADHD-200 datasets, to realize the comparison and avoid heterogeneity, parameters including the Repetition Time (TR), Echo Time (TE), voxel size, and scanner type between these two datasets are similar enough which can be utilized in deep neural network simultaneously. The detailed information of these parameters is listed in Table S2, S3. Furthermore, all datasets included are anonymous, with no protected health information included.

### Data preprocessing

The datasets used in this study were derived from preprocessed fMRI data sourced from ABIDE and ADHD-200 databases [29, 30], provided in NIFTI format. Motion correction was performed using AFNI 3dTshift tool for slice timing correction and AFNI 3dvolreg tool for head motion correction. Additionally, a conventional nuisance regression pipeline was applied consistently across both datasets. Denoising was carried out by regressing out the estimated head motion parameters. Signals from white matter and cerebrospinal fluid were also regressed out to further reduce physiological noise. Slow signal drifts were removed by incorporating linear and quadratic terms, along with low-order polynomials. Band-pass filtering (0.009–0.08 Hz) was applied to retain low-frequency fluctuations within a consistent frequency range across datasets. Finally, spatial normalization to the MNI152 standard space was performed using FSL. A two-stage registration was employed: functional images were first coregistered to each participant’s high-resolution structural image using FSL’s FLIRT, followed by normalization to the standard template using the transformation derived from the individual’s anatomical image. Spatial normalization ensured consistent anatomical localization across all participants, while preserving individual morphological variations in cortical patterning.

Following data preprocessing, multiple quality assessment methods were employed to evaluate fMRI images prior to model training. Based on the results of these metrics, images that identified as high-quality were retained, while those of low quality were excluded. Head motion quality control was implemented using a data distribution-based adaptive thresholding strategy. Specifically, thresholds for motion-related metrics, including mean framewise displacement and the number of motion-corrupted frames were adaptively determined for each dataset by setting them at the 80th or 85th percentile of their respective distributions. In addition, thresholds for translation and rotation motion parameters were determined using a composite criterion: the stricter of either the 90th percentile of the dataset or predefined absolute limits (3.0 mm for translation and 2.0 degrees for rotation). These approaches implemented an adaptive threshold framework grounded in data distribution for comprehensive quality control.

Participants data that met the established criteria were retained for further analysis. For each individual, the preprocessed 4D NIFTI volume was averaged along the time axis and systematically converted into ten 2D axial slices. Slice selection was guided by key neuroanatomical regions, including inferior cerebellum, superior cerebellum, brainstem, inferior temporal lobe, striatum and thalamus, superior temporal lobe, parietal-occipital junction, inferior frontal gyrus, middle frontal gyrus, and superior frontal gyrus. These regions were identified with reference to standardized brain atlases. Consequently, the resulting slices correspond to homologous brain regions across subjects, while retaining biologically meaningful inter-individual differences based on spatial normalization. The three-dimensional MNI coordinates of each extracted slice were systematically recorded.

### Data splitting

For each multi-site dataset, one or several specific sites were proportionally selected based on their data volume. The data from these selected sites were then held out to serve as the independent external testing set for evaluating cross-site generalizability. The details about the number of images among different sites and the selected external testing set are shown in Table S4. Furthermore, the remaining data underwent partitioning into training and validation set, preserving a proportion of 4:1 based on a five-fold cross-validation strategy. Training set and validation set were employed for model training, hyperparameter tuning, and internal validation. Testing set was employed for external validation. Moreover, considering the demographic characteristics and in order to avoid confounding, the external testing set was categorized by subgroups for further data analysis. For age groups, we categorized participants into 0–8 years old, 9–12 years old, 13–17 years old, 18–26 years old, and an unknown group based on the evolving neurodevelopmental stages of ADHD and ASD. For gender group, we divided participants into female, male, and an unknown group. Table S5 indicates the number of total images and images among subgroups (age, gender) in training set, validation set and testing set, as well as the total dataset. Given the inconsistent age ranges between the ABIDE and ADHD-200 datasets, that ADHD-200 dataset spanning 0–60 years old and ABIDE participants spanning 0–26 years old. The participants aged between 26 and 60 years old were excluded to establish comparable age distributions for subsequent analysis.

### Model architecture

We selected the following representative ImageNet pre-trained neural networks for comparison: ResNet18 [31], Xception [32], MobileNet [33], and AlexNet [34]. The weights in most layers were retained, while new layers were added or modified to the end of the structure. Dense layers were adapted to classify into five classes across all pre-trained neural networks. The input shapes were kept consistent with the original images if the models allow. The input shape of the 2D fMRI images and activation function of each architecture are listed in Table S6.

Furthermore, considering the structural resemblance and internal intricacy of the images, we opted to construct a neural network architecture based on an integration of multi-scale feature extraction, residual learning, and attention mechanisms to improve discriminative capability for complex brain structural and functional patterns called ADBrainNet. Our proposed ADBrainNet incorporates four key design innovations. Firstly, multi-scale analysis is achieved within each core Enhanced Residual Block through parallel convolutional branches of varying kernel sizes (1 × 1, 3 × 3, and 5 × 5), enabling simultaneous capture of local details and broader contextual patterns. Secondly, a channel attention mechanism (SENetLayer) is integrated to adaptively recalibrate feature responses by modeling inter-channel dependencies, thereby emphasizing informative features. Thirdly, a progressive five-stage hierarchical structure gradually abstracts and refines features through successive down sampling and bottleneck compression, promoting a generalized representation. Finally, residual shortcut connections within each block stabilize training and mitigate gradient vanishing, facilitating deeper network optimization.

**Fig. 2:**
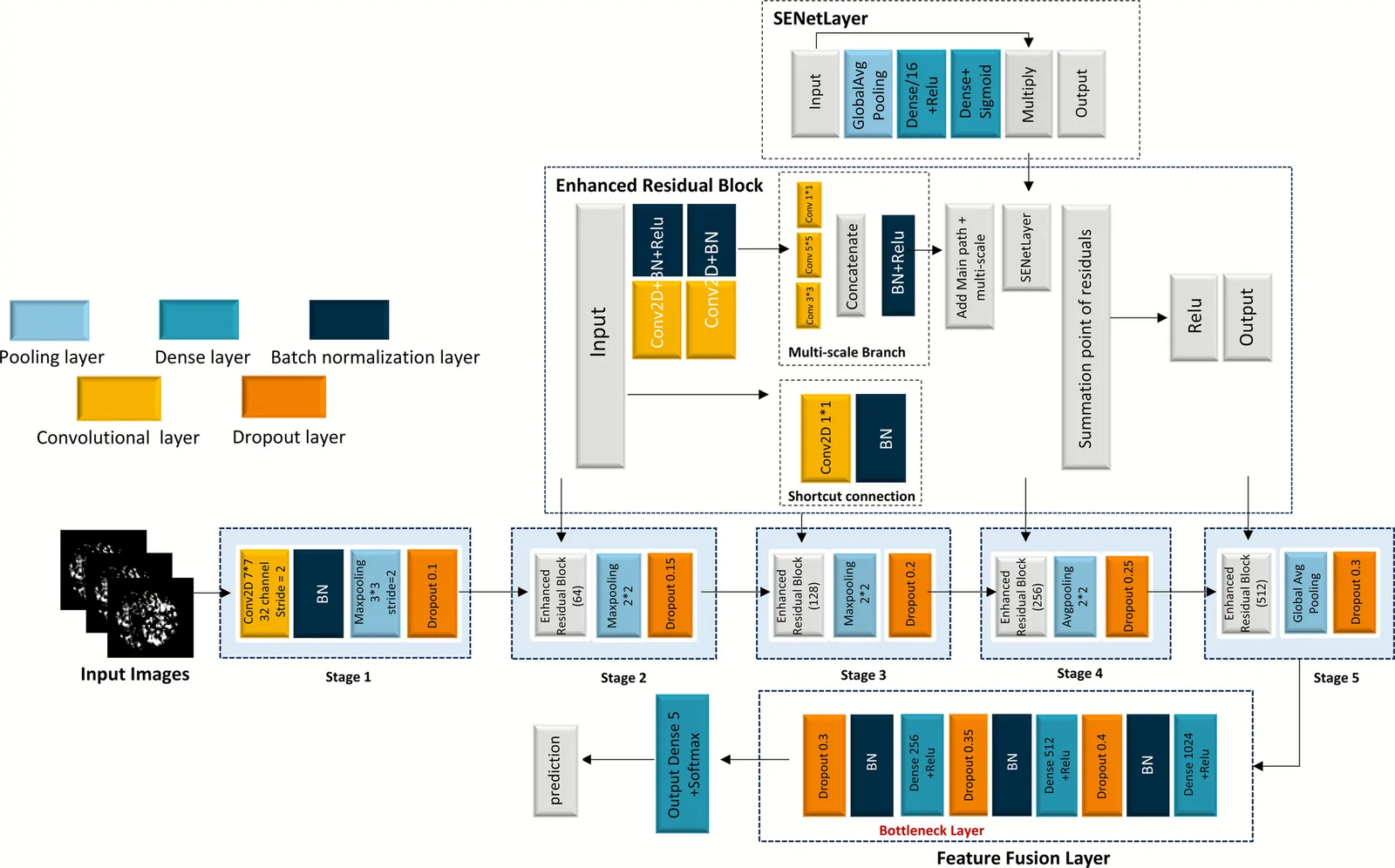
The detailed architecture of ADBrainNet

ADBrainNet architecture begins with a high-resolution feature extraction stage using a 7 × 7 convolutional kernel for a larger initial receptive field. This is followed by stages dedicated to multi-scale learning, intermediate abstraction, and high-level abstraction (Enhanced Residual Block with 64, 128, and 256 filters, respectively). A final feature refinement stage (Enhanced Residual Block with 512 filters) precedes the classification module. Spatial pooling is strategically applied using max and average pooling to reduce dimensionality while preserving essential information. Regularization techniques, including L2 weight decay and progressively increased dropout rates [35], are employed throughout to prevent overfitting. The network culminates in a global average pooling layer and a sequence of fully connected layers for final prediction. Detailed ADBrainNet architectural parameters, including filter counts, kernel sizes, and layer configurations, are provided in Fig. 2.

### Training strategy

The experiments were carried out in a Google Colab notebook based on Python version 3.10.12. The cloud environment featured a tensor processing unit (TPU), a T4 GPU runtime, and an Intel Core i5 processor boasting 12.67 GB of RAM. The model establishment, model training, model evaluation, and numerical computation use the Keras API with TensorFlow backend.

Considering the difficulty of the five-class classification task, we employed a dynamic learning rate scheduler. The initial learning rate was set to 0.002. If the validation loss failed to decrease for 5 consecutive epochs, the learning rate was reduced by a factor of 0.2 to ensure the model optimizes in the right direction. Furthermore, we set up the early stopping point when the validation loss does not decrease after 20 epochs. We performed the initial 100 epochs and batch size as 64 for each training process and defined the values of steps for each epoch as the sample size divide batch size on the training and validation sets, employing the Adam optimizer. For the loss function, we applied categorical cross-entropy function. Five-fold cross-validation were performed for the training set and validation set. The five splitting have same proportion to make the result compared.

To mitigate class imbalance and enhance model generalizability, we employed a class weighting scheme alongside differentiated data augmentation strategies on the training set. We set up the class weight for ADHD combined, ADHD inattentive, ADHD hyperactive/impulsive, ASD, and neurotypical categories with a proportion of 2.0: 3.5: 2.5: 1.5: 1.5 based on their general image number respectively. Furthermore, during every fold, different augmentation strategies and factors were proposed on each class to mitigate the imbalance after the dataset was divided. We performed two types of strategy including geometric transformations (rotation, horizontal flip, and vertical flip) and color enhancements (brightness, contrast, sharpness, and color balance). The detailed parameters and factors of data augmentation are listed in Table S7. Throughout the training process, we ensured datasets shuffling to enhance model robustness.

### Model evaluation

Five indicators were selected to evaluate the effectiveness of these models. Accuracy, denoting the proportion of samples accurately classified by the model in a categorical classification scenario. Precision evaluates the ratio of true positive predictions out of all the samples predicted as positive by the model. Recall evaluates the sensitivity of the model. The harmonic mean of recall and precision is indicated by F1 score which considers the precision as well as completeness of the model. AUC in a five-class scenario evaluates the model’s ability to distinguish between different classes by performing one-vs-rest binary comparisons for each class, generating multiple ROC curves, and calculating the area under each curve. For each performance metric, the result is reported as the mean ± standard deviation (SD) across five-fold cross-validation. The evaluation of each subgroup includes accuracy, precision, recall, and F1 score. For each category, the evaluation focuses on precision, recall, and F1 score.

We conducted statistical analysis to evaluate the significance of performance differences among the five deep learning models. Given the nature of our five-fold cross-validation data, we employed the Friedman test, a non-parametric method suitable for comparing three or more related samples. This test determines whether there are statistically significant differences in model performance across all evaluation metrics. For post-hoc analysis following significant Friedman results, we utilized the Nemenyi test to perform pairwise comparisons between models. This method controls for multiple comparison errors while identifying which specific model pairs differ significantly. Statistical significance was set at p < 0.05. Furthermore, the mean ± SD prediction time per-image was derived to assess the potential of the models’ deployment on clinical devices.

LIME algorithm was proposed to generate post-hoc, sample-specific explanations. For a given input image, LIME produces a saliency map that highlights the regions which most contributed to the model’s classification decision for that specific sample. We selected a subset of test images from each class for representation, specifically choosing those on which the model had high prediction confidence (probability > 0.8). The rationale for this selection is that high-confidence predictions correspond to cases where the model is most decisive. Therefore, the corresponding explanations are less ambiguous and provide a more interpretable illustration of the features the model leverages when it is confident. Notably, we emphasized that LIME offers an exploratory interpretation of results by selecting only typical images based on established neuroscientific frameworks. Due to the constraints of our sample and the post-hoc nature of interpretability methods, these associations are speculative and intended to guide future research. We set up the number_samples as 2000, top_labels as 1, and number_features as 5 for LIME considering the medical images characteristics. Following the application of LIME algorithm on individual 2D slices, the resulting feature importance maps could systematically project back to their corresponding MNI coordinates within the standardized anatomical space based on the previous spatial normalization.

## Results

### General model performance on the independent external testing set

We compared the performance with the baseline models and our preprocessed models incorporating both class weight and data augmentation addressing class imbalance on the external testing set. Table S8 and Table S9 summarize the performance of baseline models on the external testing set without addressing class imbalance. Table S8 presents the overall metrics (accuracy, precision, recall, and F1-score) for each fold and the mean ± SD through the five-fold cross-validation. Table S9 reports the per-category performance metrics (precision, recall, and F1-score). These results demonstrated a pronounced majority-class bias in the baseline model. Its performance was almost exclusively limited to the neurotypical class, resulting in inflated overall metrics that masked its near-zero predictive validity for the ASD and ADHD subgroups.

Among the preprocessed models, ADBrainNet outperformed other deep neural networks and achieved best performance with mean (± SD) accuracy, precision, recall, and F1 score of 61.87% (± 5.59%), 65.72% (± 6.98%), 61.87% (± 5.59%), and 62.50% (± 5.78%) respectively through five-fold cross-validation on the independent external testing set. This indicated that the class imbalance issue was effectively mitigated compared with the baseline models. AlexNet and Xception trailed ADBrainNet, attaining the mean (± SD) accuracy of 50.29% (± 3.14%) and 45.53% (± 3.03%) on the external testing set respectively. ResNet18 and MobileNet exhibited a sign of overfitting, likely attributed to single feature extraction from the training set, evidenced by their diminishing accuracy and seem not appropriate for this mission. Their overall accuracy was predominantly attributed to the high classification performance on neurotypical participants, whereas the predictive accuracy for the ASD and ADHD groups was relatively lower. Tables 1 and 2 summarize the accuracy, precision, recall, and F1 score obtained from the final models for each fold, along with the mean ± SD across the five-fold cross-validation on the external testing set. Class imbalance was addressed during the computation of these metrics. Moreover, the detailed performance metrics (accuracy, precision, recall, and F1 score) from the five-fold cross-validation on the internal validation set are provided in Table S10. These metrics were derived from the model checkpoint with the lowest validation loss in each fold, ensuring transparency in model development and performance consistency.

**Table 1:** Performance metrics (accuracy, precision, recall, and F1 score) on the external testing set for each model per fold, trained with class weighting and data augmentation to mitigate class imbalance

Model	Accuracy (%)	Precision (%)	Recall (%)	F1 score (%)
ADBrainNet				
Fold1	55.45	60.15	55.45	57.15
Fold2	70.52	76.14	70.52	72.21
Fold3	60.52	61.76	60.52	60.48
Fold4	63.25	69.70	63.25	62.74
Fold5	59.61	60.85	59.61	59.90
AlexNet				
Fold1	51.43	44.65	51.43	45.40
Fold2	50.65	52.38	50.65	49.52
Fold3	52.21	47.08	52.21	46.45
Fold4	44.81	34.13	44.81	36.20
Fold5	52.34	53.67	52.34	49.88
MobileNet				
Fold1	34.29	56.04	34.29	35.95
Fold2	47.79	59.45	47.79	47.28
Fold3	48.18	57.35	48.18	44.87
Fold4	33.38	57.95	33.38	33.67
Fold5	34.68	55.10	34.68	29.01
ResNet18				
Fold1	33.51	56.15	33.51	28.34
Fold2	40.00	58.64	40.00	40.29
Fold3	22.86	53.54	22.86	20.31
Fold4	42.73	55.69	42.73	45.94
Fold5	44.81	53.71	44.81	45.99
Xception				
Fold1	47.27	57.77	47.27	50.22
Fold2	41.17	54.50	41.17	45.65
Fold3	45.97	57.71	45.97	48.37
Fold4	44.16	55.77	44.16	47.69
Fold5	49.09	57.54	49.09	50.95

**Table 2:** Model performance comparison showcasing the accuracy, precision, recall, and F1 score (mean ± SD), obtained from five-fold cross-validation on the external testing set

Model	Accuracy (%, rank)	Precision (%)	Recall (%)	F1 score (%)
ADBrainNet	61.87 ± 5.59 (1)	65.72 ± 6.98	61.87 ± 5.59	62.50 ± 5.78
AlexNet	50.29 ± 3.14 (2)	46.38 ± 7.79	50.29 ± 3.14	45.49 ± 5.54
Xception	45.53 ± 3.03 (3)	56.66 ± 1.46	45.53 ± 3.03	48.58 ± 2.11
MobileNet	39.66 ± 7.61 (4)	57.18 ± 1.69	39.66 ± 7.61	38.16 ± 7.70
ResNet18	36.78 ± 8.87 (5)	55.55 ± 2.08	36.78 ± 8.87	36.17 ± 11.42

**Fig. 3:**
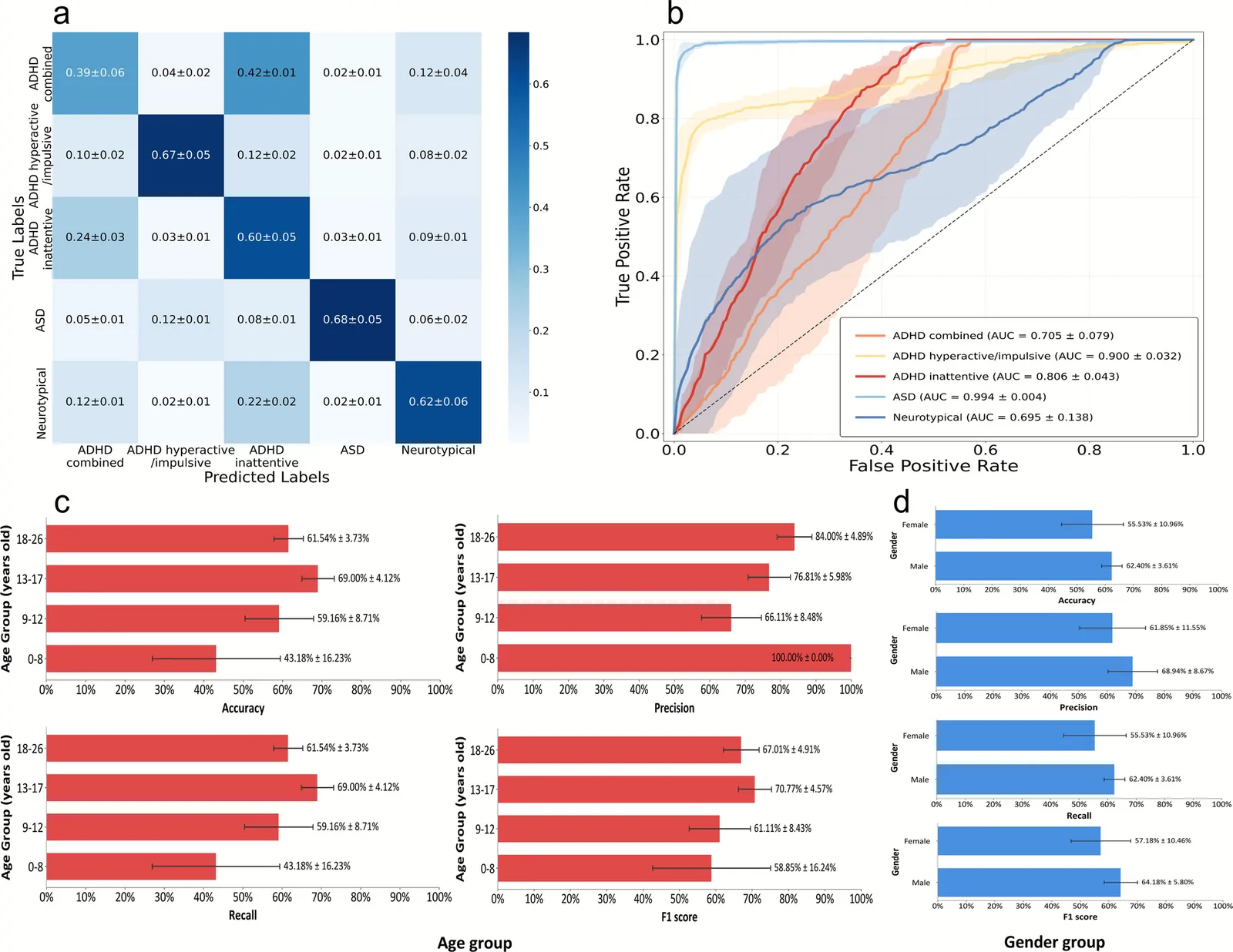
ADBrainNet performance on the external testing set. a, confusion matrix between the predicted labels and true labels on the external testing set with mean ± SD normalized values across five-fold cross-validation. b, Receiver Operating Characteristic (ROC) curves from five-fold cross-validation analysis on the external testing set of ADBrainNet. Solid lines represent the mean ROC curves for each category across all folds, while shaded regions indicate the SD across the five folds. c, performance metrics across age groups on the external testing set of ADBrainNet. Bars represent mean values of accuracy, precision, recall, and F1 score, with error bars indicating SD from five-fold cross-validation. d, performance metrics across gender groups on the external testing set of ADBrainNet. Bars represent mean values of accuracy, precision, recall, and F1 score, with error bars indicating SD from five-fold cross-validation

Moreover, ADBrainNet achieved AUC scores reported as mean ± SD: 0.70 ± 0.08 for the ADHD combined class, 0.90 ± 0.03 for the ADHD hyperactive/impulsive class, 0.81 ± 0.04 for the ADHD inattentive class, 0.99 ± 0.01 for the ASD class, and 0.70 ± 0.14 for the neurotypical class on the external testing set. The results between true labels and predicted labels of ADBrainNet on the external testing set are indicated by the confusion matrix, with mean ± SD normalized values derived from five-fold cross-validation (Fig. 3). It indicated that there was relatively more confusion in predicting between ADHD combined and ADHD inattentive categories which should be addressed. Table S11 shows the per-class metrics (precision, recall, and F1 score) of the final models on the external testing set for each fold.

Statistical analysis utilizing the Friedman test revealed significant differences among the five models across all evaluation metrics (all p < 0.05) on the external testing set. ADBrainNet consistently achieved the best performance, ranking first in all four metrics. Post-hoc Nemenyi tests also revealed that ADBrainNet outperformed other models significantly in pairwise comparison on the external testing set. Specifically, ADBrainNet surpassed MobileNet and ResNet18 in three metrics (accuracy, recall, and F1 score), and AlexNet in precision (p < 0.005). However, no significant differences were observed between ADBrainNet and Xception across all metrics. The detailed results of Friedman test and Post-hoc Nemenyi tests on the external testing set are shown in Tables 3 and 4. All evidence indicated that ADBrainNet was the model with best performance generatively.

**Table 3:** Friedman test results comparing the performance of five models across four evaluation metrics on the external testing set

Metrics	Best model	Friedman χ^2^ (4)	*P* value
Accuracy	ADBrainNet	17.44	< 0.01*
Precision	ADBrainNet	17.12	< 0.01*
Recall	ADBrainNet	17.44	< 0.01*
F1 score	ADBrainNet	15.52	< 0.01*

Significance levels are indicated as follows: *p < 0.01

**Table 4:** Post-hoc Nemenyi test results for pairwise comparisons between ADBrainNet and other models across four evaluation metrics on the external testing set

Comparison	Accuracy (*P* value)	Precision (*P* value)	Recall (*P* value)	F1 score (*P* value)
ADBrainNet vs AlexNet	0.86	< 0.01**	0.86	0.27
ADBrainNet vs MobileNet	0.02*	0.37	0.02*	0.01*
ADBrainNet vs Xception	0.12	0.50	0.12	0.63
ADBrainNet vs ResNet18	< 0.01**	0.07	< 0.01**	< 0.01**

Significance levels are indicated as follows: *p < 0.05, **p < 0.01

In terms of computational efficiency, ADBrainNet achieved the shortest mean ± SD prediction time per-image (34.21 ± 0.61 ms) among the compared deep neural networks on the external testing set, followed by ResNet18. Combined with its superior evaluation metrics, ADBrainNet demonstrated the best overall performance, achieving an optimal balance between speed and accuracy. Table 5 details the number of parameters and per-image prediction time for different deep neural works on the external testing set. It presents these values for each fold, along with the mean ± SD across the five-fold cross-validation.

**Table 5:** Model parameters and prediction time per-image (mean ± SD) for different neural networks, evaluated across five-fold cross-validation on the external testing set

Model	Number of parameters (million)	Average prediction time per image (millisecond)
ADBrainNet	9.27	34.21 ± 0.61
AlexNet	4.06	39.58 ± 11.03
MobileNet	3.23	86.43 ± 25.26
ResNet18	11.32	38.85 ± 1.71
Xception	21.91	92.40 ± 1.18

Subgroup analysis reveals distinct performance patterns across age groups and gender groups on the external testing set (Fig. 3, Table S12). The highest mean accuracy (69.00% ± 4.12%), recall (69.00% ± 4.12%), and F1 score (70.77% ± 4.57%) were observed in the 13–17 age group. The 0–8 age group demonstrated perfect precision, with all test samples correctly predicted. In the gender-based analysis, ADBrainNet demonstrated consistently higher performance for male participants (accuracy: 62.40% ± 3.61%, precision: 68.94% ± 8.67%, recall: 62.40% ± 3.61%, and F1-score: 64.18% ± 5.80%) compared to female participants across all metrics. While predictive capability was confirmed for both genders, a measurable performance disparity favoring males was observed within this cohort.

### LIME used for explainable artificial intelligence

**Fig. 4:**
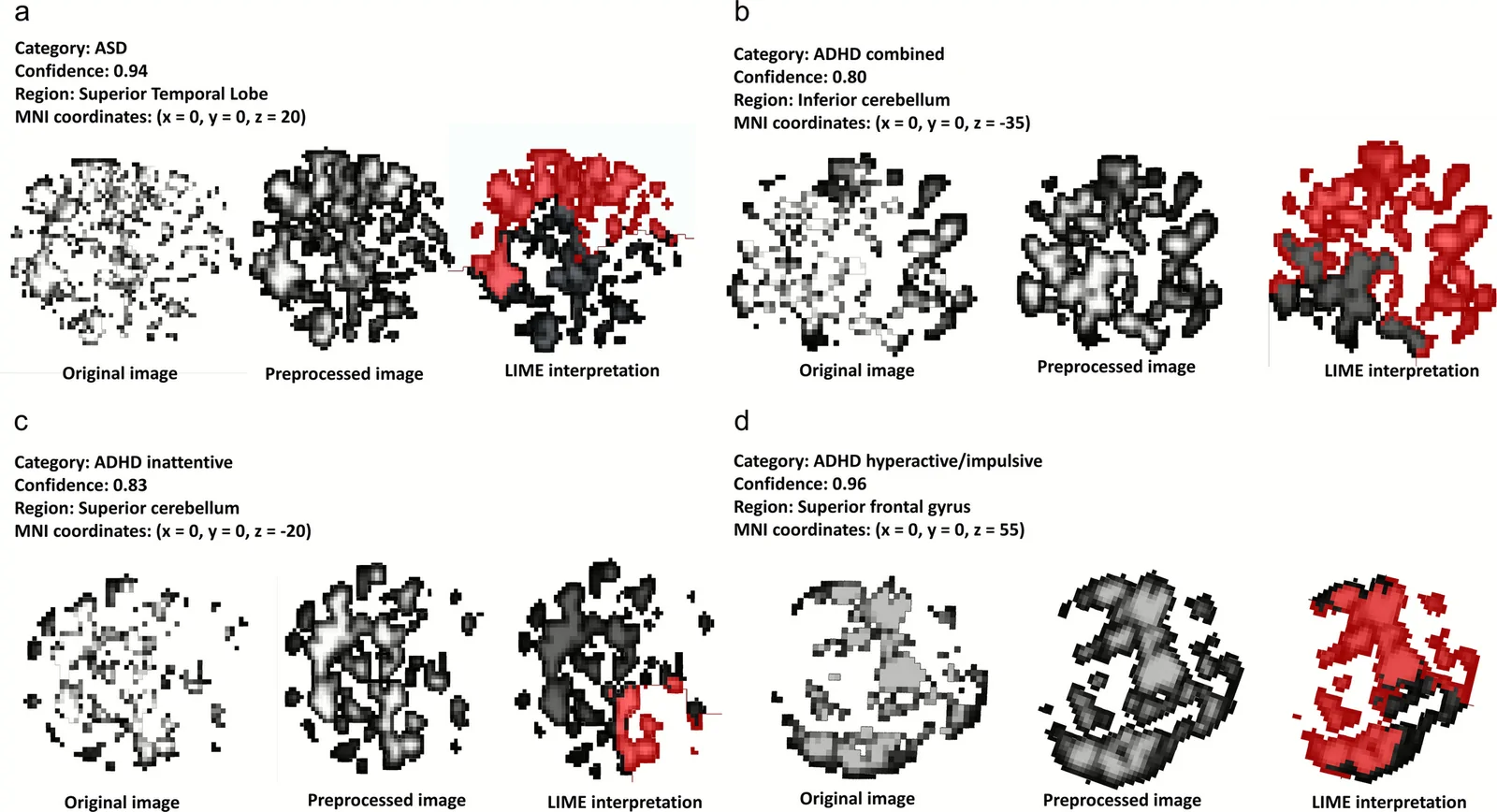
Explainability analysis of typical high-confidence images using LIME on ADBrainNet predictions. From left to right: the original input image; a representative perturbed sample generated by LIME to probe the model’s local behavior; and the final saliency map with the most contributory image region (highlighted in red) overlaid on the original slice. a, visualization of the LIME explanation process for a selected ASD 2D axial slice through the superior temporal lobe (MNI coordinates: 0, 0, 20; confidence: 0.94). b, visualization of the LIME explanation process for a selected ADHD combined 2D axial slice through the inferior cerebellum (MNI coordinates: 0, 0, −35; confidence: 0.80). c, visualization of the LIME explanation process for a selected ADHD inattentive 2D axial slice through the superior cerebellum (MNI coordinates: 0, 0, −20; confidence: 0.83). d, visualization of the LIME explanation process for a selected ADHD hyperactive/impulsive 2D axial slice through the superior frontal gyrus (MNI coordinates: 0, 0, 55; confidence: 0.96)

To derive interpretable insights, we applied the LIME algorithm to test selected samples with high predictive confidence (probability > 0.8). The resulting saliency maps, serving as exploratory visual explanations, including the category, confidence, region and MNI coordinates are presented in Fig. 4. It is critical to emphasize that LIME provided exploratory, sample-specific visualizations rather than definitive biomarkers. The resulting associations between image features and model output are speculative by nature, due to the post-hoc interpretation method and the limited sample size, particularly within underrepresented subgroups. These visualizations are intended solely to generate hypotheses for future research within established neuroscientific contexts.

For ASD, the LIME primarily indicated coverage over the left superior temporal lobe. The coverage included the posterior part of the left superior temporal gyrus (encompassing Wernicke’s area) and the left superior temporal sulcus. These regions are described as an integrative hub in the literature for social information processing like speech, lip movement, facial expressions, and prosody [36]. For ADHD inattentive, the LIME identified the brainstem (including reticular formation and locus coeruleus), and the superior cerebellum. The brainstem is considered as a physiological basis for attention maintenance difficulties and susceptibility to fatigue, while the superior cerebellum is a key region associated with time perception deficits and reduced cognitive fluency [37]. For ADHD combined, the LIME indicated the inferior cerebellum, particularly the cerebellar tonsillar region and the structures at the base of the posterior cranial fossa. It is noteworthy that these regions are implicated in sensory filtering and intracranial pressure regulation according to established literature [38]. For ADHD hyperactive/impulsive, the LIME mask localized to the inferior portion of the superior frontal gyrus, covering the supplementary motor area and anterior cingulate cortex. These regions are widely regarded in the literature as the highest-order command centers for behavioral control [39].

## Discussion

We presented a lightweight deep neural network, ADBrainNet, which effectively distinguished five classes including ADHD combined, ADHD hyperactive/impulsive, ADHD inattentive, ASD, and neurotypical individuals using resting-state fMRI images. Our findings demonstrated that ADBrainNet outperformed other ImageNet pre-trained models, achieving the best performance with mean (± SD) accuracy, precision, recall, and F1 score of 61.87% (± 5.59%), 65.72% (± 6.98%), 61.87% (± 5.59%), and 62.50% (± 5.78%) respectively across five-fold cross-validation on the external testing set. This five-class classification task was more complex than previous studies reported by Mozhdefarahbakhsh et al., who achieved an accuracy of 0.92 on the binary classification task without a dedicated testing dataset [14]. Furthermore, our five-class classification task showed significant improvement over the short-term binary diagnosis between ASD and ADHD, with mean accuracies of 0.687 ± 0.005 and 0.650 ± 0.014 respectively, as reported by Huang et al. [15]. ADBrainNet’s best performance in precision was especially relevant for medical applications, where minimizing false positives is crucial to avoid unnecessary clinical interventions. Moreover, the confusion matrix revealed a tendency for misclassification between ADHD combined and ADHD inattentive subtypes. One exploratory interpretation is that when attention deficits co-occur with hyperactive or impulsive symptoms, the hyperactivity component may be overshadowed, potentially contributing to the observed misclassification. Alternatively, the brain may more readily capture signals related to attention deficits, while the more transient and short-duration signals of hyperactivity/impulsivity may harder to detect. Therefore, a promising direction for future research could involve conducting a large-scale binary classification analysis between ADHD combined and ADHD inattentive type, potentially assists by multimodal EEG signals, to capture more subtle or rare hyperactive/impulsive features. Subsequently, these refined discriminative capabilities could be integrated into the overall five-class classification framework.

The Friedman test results (all p < 0.05) indicated that the observed performance differences among the five models are highly unlikely to be due to random chance. This strong statistical evidence validated our experimental design and suggested that architectural differences among the models impacted their classification performance on brain MRI data. ADBrainNet’s achievement of the best average rank first across all metrics was particularly noteworthy. The convergence of these results across multiple performance metrics, each capturing a distinct aspect of classification quality strengthens the evidence for ADBrainNet’s robustness. The Nemenyi post-hoc tests revealed nuanced patterns in model comparisons. The consistent and significant outperformance of ADBrainNet over MobileNet and ResNet18 across multiple evaluation metrics suggested that these general-purpose architectures may be suboptimal for addressing the specific complexities of neuroimaging data. Interestingly, the lack of significant difference between ADBrainNet and Xception, despite ADBrainNet’s consistent numerical superiority, warrants further investigation with larger datasets to determine if this represents a genuine performance equivalence or a statistical power limitation. Moreover, our ADBrainNet attained the shortest average prediction time per-image, reflecting its optimized architecture and effective parameter utilization. This efficiency did not come at the expense of accuracy, suggesting that the integration of multi-scale feature extraction and attention mechanisms may successfully balance representational capacity with computational cost. Although the fact that ADBrainNet achieved the shortest inference time despite its slight larger parameter count than some baseline models suggested a more computationally efficient architecture, like featuring higher parallelism and attention mechanism. Such efficiency facilitates faster processing, which was a desirable characteristic for potential deployment in real-time or batch processing scenarios within clinical workflows.

The model subgroup analysis demonstrated high accuracy and robust predictive performance across three key metrics in the 13–17 and 18–26 age groups. Meanwhile, the 0–8 age group exhibited an exceptionally high level of precision. This implied that in actual clinical practice, applying our model to the 0–8 age group may result in an extremely low probability of missed diagnosis. Finally, the 9–12 age group exhibited lower scores across the four evaluation metrics, suggesting that more complex or heterogeneous features may characterize pre-adolescence patients. This phenomenon likely stems from the critically dynamic neurodevelopmental phase characteristic of this age range. During the pre-adolescent transition, the brain undergoes extensive synaptic reorganization and network refinement. This period is marked by heightened inter-individual heterogeneity in functional connectivity patterns. Consequently, the distinct neural signatures that typically differentiate neurodevelopmental disorders from normative development may become less demarcated, as atypical developmental trajectories exhibit greater variability and overlap with typical ones [40]. In the future, we aim to integrate medical history and EEG data with analysis on a larger cohort of pre-adolescence patients to further identify discriminative features and improve classification performance in this age range. Gender-based analysis revealed a consistent trend of better model performance for male participants across all evaluation metrics. It may suggest the female phenotype involve more subtle or heterogeneous neural alterations that are inherently more challenging for classification algorithms to capture [41].

Beyond demographic factors, we sought to gain a preliminary, interpretable insight into the neuroanatomical basis of the model’s classifications via LIME. It is important to note that this analysis is inherently exploratory. The interpretations derived from LIME are post-hoc and should be viewed as generating hypotheses about potential neural correlates, especially given our sample size constraints. The regions highlighted by LIME in the ASD group, particularly the left superior temporal lobe, may point to the potential involvement of associated neural systems in the model’s classification decision. This area is a key hub for social-auditory processing, supporting functions such as interpreting eye contact and social cues, as well as language processing, which are often abnormal in ASD. This suggests that the model may have leveraged features related to the neural substrates underlying these specific social-communicative impairments, a core domain of the condition [42]. In the prevailing pathophysiological models of ADHD attentive subtypes, dysregulation of the brainstem is thought to underlie unstable cortical arousal, while impairment in the superior cerebellum has been linked to symptoms of disorganization and attentional drift. Our LIME findings, which highlighted these very regions, may therefore be seen as resonating with these theoretical frameworks [43]. The inferior cerebellum and posterior cranial fossa regions highlighted by LIME in the ADHD combined subgroup are of particular interest. These areas are implicated in sensory filtering and brainstem modulation, processes relevant to core ADHD symptoms. We therefore cautiously posit that the model’s reliance on these regions may reflect a neural substrate where deficits in filtering and arousal regulation could converge, potentially contributing to the complex symptom profile characteristic of the combined subtype, although this specific causal pathway remains to be directly tested [44]. Within the inferior portion of the superior frontal gyrus, the supplementary motor area is crucial for motor programming and inhibition, and the anterior cingulate cortex for conflict monitoring and motivational evaluation. The convergence between our LIME findings in these regions and the classic symptoms of ADHD hyperactive/impulsive (e.g., impaired inhibition, impulsivity) suggested a plausible neuroanatomical substrate for the disorder. We hypothesized that variations in this fronto-cingulate circuit may be associated with the expression of hyperactive and impulsive behaviors [45].

Improving interpretability in artificial intelligence for medical diagnostics serves to strengthen the confidence and reliance of healthcare practitioners in AI systems. Our analysis of the model’s behavior suggested a systematic approach to processing fMRI data, with indications that it prioritized features related to the overall brain silhouette, edges, and internal structural contours across different orientations. For an exploratory interpretation of the model’s behavior, we applied the LIME algorithm. The resulting feature visualizations were illustrative, based on analysis of representative test samples, and were intended to generate hypotheses about the image features that may inform the model’s decisions. We proposed that such computational explanations could, in future applications, may offer a complementary perspective for clinical research. By highlighting regions that the model finds informative, it might prompt clinicians to consider or re-evaluate corresponding areas of interest in fMRI scans. Ultimately, if validated in larger and more diverse cohorts, such tools have the potential to contribute to diagnostic assessments, which could help reduce diagnostic uncertainty and its associated psychological burden on patients and families.

This study does present several limitations and potential avenues for future research. Considering the clinical reality of comorbid ADHD and ASD, the model needs to be adapted for multi-label classification tasks. This entails leveraging techniques such as GAN and image synthesis to augment training data with multiple labels [46–48]. Based on the different performance between different age and gender groups, stratifying the analysis and trains by each group of these variables could reveal potential subgroup differences and assure the large database can be applied on corresponding subgroups of different models. Furthermore, the model’s lightweight architecture and maintained accuracy may suggest a potential advantage for deployment in settings with limited computational resources, such as mobile or point‑of‑care health applications. Future work will focus on further model optimization and validation in prospective clinical environments to assess its practical utility in assisting early detection and assist-monitoring. Moreover, the explanations derived from LIME are inherently local and can be sensitive to parameter choices (e.g., feature segmentation); therefore, the insights generated from our analysis of a limited, curated subset of samples should be viewed as illustrative and exploratory, not as stable or generalizable conclusions. To strengthen the robustness of our interpretations, future work should employ more stable interpretable frameworks and adopt quantitative metrics (e.g., faithfulness) to evaluate explanation reliability systematically. Furthermore, this study stems from the limited sample size, most notably within the ADHD hyperactive/impulsive subgroup, which inherently creates a class imbalance. To address this, we implemented comprehensive data augmentation and static class weighting during training. While this strategy helped mitigate the overall imbalance, its fixed nature may not fully capture the nuanced learning challenges posed by the very small number of samples in the minority class. Therefore, we plan to explicitly investigate advanced reweighting strategies in future work. Incorporating dynamic, sample-aware reweighting algorithms will be a key focus to further refine the model’s learning focus, especially on challenging or rare samples within already minority classes. Finally, integrating patient-specific characteristics and medical histories, and EEG signals into the neural network alongside resting-state fMRI images as multimodal deep neural network may enhance the model’s predictive accuracy and clinical utility.

## Conclusion

ADBrainNet is designed to assist in the differentiation among ADHD combined, ADHD hyperactive/impulsive, ADHD inattentive, ASD, and neurotypical individuals. Its lightweight architecture and interpretable design are intended to facilitate potential clinical application by providing efficient and explainable predictions. This tool could help reduce the likelihood of misdiagnosis between ASD and ADHD in principle. In turn, this might contribute to more appropriate treatment planning and efficient use of clinical resources, although such outcomes require formal evaluation in real-world healthcare settings. Furthermore, the interpretability of the model aims to make the diagnostic decision process more transparent. This transparency could potentially help clinicians understand the basis of the model’s predictions, which may support diagnostic reasoning. Ultimately, if validated and integrated thoughtfully, such tools could help reduce diagnostic uncertainty, thereby potentially lessening patient and family distress associated with ambiguous diagnoses.

## Supplementary Information

Below is the link to the electronic supplementary material.

Supplementary file1 (DOCX 107 KB)

## Data Availability

Data used in this study are all obtained from NITRC program (https://www.nitrc.org/).

## Code availability

Python script of ADBrainNet framework is available on Github (https://github.com/YIXinyao1/ADBrainNet/tree/main).
