# Supplementary material for: ADBrainNet: a deep neural network for Autism Spectrum Disorder (ASD) and Attention Deficit and Hyperactivity Disorder (ADHD) classification using resting-state fMRI images based on explainable artificial intelligence
Source: Med Biol Eng Comput. 2026 Mar 5;64(5):1609–22. doi: 10.1007/s11517-026-03530-2 (PMC13695582; doi:10.1007/s11517-026-03530-2)
Supplement: Supplementary file 1 [file 11517_2026_3530_MOESM1_ESM.docx]

**Table of Contents**

**Table S1** Demographic distribution between five classes (ADHD combined, ADHD hyperactive/impulsive, ADHD inattentive, ASD, and neurotypical), including age, gender, and chirality distributions (P-value by ANOVA and Chi-Squared Test)

**Table S2** Detailed parameters of images derived from ABIDE dataset, including the TR, TE, Voxel Size and Scanner type

**Table S3** Detailed parameters of images derived from ADHD-200 dataset, including the TR, TE, Voxel Size and Scanner type

**Table S4** Sample sizes by site and diagnostic category. The number of participants from each scanning site is stratified by diagnostic group. Sites selected for the external testing set are indicated in parentheses

**Table S5** Distribution of final image counts for each subgroup (age, gender) in training set, validation set and testing set, as well as the total dataset

**Table S6** The input shape, newly added/modified layers and activation function incorporated into each pre-trained deep neural network and ADBrainNet

**Table S7** Details of class-specific data augmentation strategy

**Table S8** The detailed performance of the baseline models without class imbalance mitigation on the external testing set. Metrics including accuracy, precision, recall, and F1-score are reported for each fold, along with the mean ± SD across the five-fold cross-validation

**Table S9** Per-class performance (precision, recall, and F1 score, in %) of the baseline models on the external testing set without class imbalance mitigation

**Table S10** The detailed performance of the final models on the internal validation sets using class weighting and data augmentation to mitigate class imbalance. Metrics including accuracy, precision, recall, and F1 score are reported for each fold, along with the mean ± SD from five-fold cross-validation

**Table S11** Per-class performance (precision, recall, and F1 score, in %) of the final models on the external testing set after class imbalance mitigation

**Table S12** The detailed performance of ADBrainNet across different subgroups (age, gender) on the external testing set. Metrics including accuracy, precision, recall, and F1 score are reported for each fold, along with the mean ± SD from the five-fold cross-validation

**Table S1** Demographic distribution between five classes (ADHD combined, ADHD hyperactive/impulsive, ADHD inattentive, ASD, and neurotypical), including age, gender, and chirality distributions (P-value by ANOVA and Chi-Squared Test)

| **Characteristic** | **Neurotypical**  **(N =1,766)** | | **ASD**  **(N = 1,083)** | **Attention deficit hyperactivity disorder (ADHD)** | | | **P-value** |
| --- | --- | --- | --- | --- | --- | --- | --- |
|  |  |  |  | **ADHD**  **inattentive (N = 137)** | **ADHD**  **hyperactive/impulsive**  **(N = 13)** | **ADHD**  **combined**  **(N = 212)** |  |
| Age (years)^a^ | | 14.30 (7.59) | 15.43 (8.65) | 11.59 (2.59) | 12.31 (4.31) | 10.78 (3.16) | < 0.001 |
| Gender | | | | | | | < 0.001 |
| Female | | 561 (31.77) | 145 (13.39) | 38 (27.74) | 2 (15.38) | 37 (17.45) |  |
| Male | | 1,205 (68.23) | 938 (86.61) | 99 (72.26) | 11 (84.62) | 174 (82.08) |  |
| Unknown | | 0 (0.00) | 0 (0.00) | 0 (0.00) | 0 (0.00) | 1 (0.47) |  |
| Chirality | | | | | | | < 0.001 |
| Left-handed | | 86 (4.87) | 109 (10.06) | 2 (1.46) | 1 (7.69) | 8 (3.77) |  |
| Right-handed | | 1,621 (91.79) | 901 (83.19) | 135 (98.54) | 11 (84.62) | 201 (94.81) |  |
| All | | 8 (0.45) | 10 (0.92) | 0 (0.00) | 1 (7.69) | 0 (0.00) |  |
| Unknown | | 51 (2.89) | 63 (5.82) | 0 (0.00) | 0 (0.00) | 3 (1.42) |  |

Numbers are n (%) unless otherwise stated. ^a^Displayed as the mean (standard deviation) of age in each group.

**Table S2** Detailed parameters of images derived from ABIDE dataset, including the TR, TE, Voxel Size and Scanner type

| **Site** | **TR (ms)** | **TE (ms)** | **Voxel Size (rs-fMRI)** | **Scanner Type** |
| --- | --- | --- | --- | --- |
| NYU | 2,000 | 15 | 3.0*3.0*4.0 | Siemens Magnetom Allegra |
| Peking | 2,000 | 30 | 3.1*3.1*3.0 | Siemens Magnetom Trio Tim |
| KKI | 2,500 | 30 | 3.0*3.0*3.0 | Philips 3T Achieva |
| OHSU | 2,500 | 30 | 3.8*3.8*3.8 | Siemens Magnetom Trio Tim |
| NeuroIMAGE | 1,960 | 40 | 3.5*3.5*3.0 | Siemens Magnetom Avanto |
| Pittsburgh | 1,500 | 29 | 3.1*3.1*4.0 | Siemens Magnetom Trio Tim |
| WashU | 2,500 | 27 | 4.0*4.0*4.0 | Siemens Magnetom Trio Tim |

**Table S3** Detailed parameters of images derived from ADHD 200 dataset, including the TR, TE, Voxel Size and Scanner type

| **Site** | **TR (ms)** | **TE (ms)** | **Voxel Size (rs-fMRI)** | **Scanner Type** |
| --- | --- | --- | --- | --- |
| Caltech | 2,000 | 30 | 3.5×3.5×3.5 | Siemens Magnetom Trio Tim |
| CMU | 2,000 | 30 | 3.0×3.0×3.0 | Siemens Magnetom Verio |
| KKI | 2,500 | 30 | 3.0×3.0×3.0 | 3 Tesla Philips Achieva |
| Leuven | 1,667 | 33 | 3.5×3.5×3.5 | 3 Tesla Philips Achieva |
| Max_Mun | 3,000 | 30 | 3.0×3.0×4.0 | Siemens Magnetom Verio |
| NYU | 2,000 | 15 | 3.0×3.0×4.0 | Siemens Magnetom Allegra |
| OHSU | 2,500 | 30 | 3.8×3.8×3.8 | Siemens Magnetom Trio Tim |
| Olin | 1,500 | 27 | 3.4×3.4×4.0 | Siemens Magnetom Allegra |
| Pitt | 1,500 | 25 | 3.1×3.1×4.0 | Siemens Magnetom Allegra |
| SBL | 2,200 | 30 | 2.75×2.75×2.72 | Philips Intera 3T |
| SDSU | 2,000 | 30 | 3.0×3.4×3.4 | GE 3T MR750 |
| Stanford | 2,000 | 30 | 3.125×3.125×4.5 | GE SIGNA 3T |
| Trinity | 2,000 | 28 | 3.0×3.0×3.5 | Philips 3T Achieva |
| UCLA | 3,000 | 28 | 3.0×3.0×4.0 | Siemens Magnetom Trio Tim |
| UM | 2,000 | 30 | 3.438×3.438×3.0 | 3 Tesla GE Signa |
| USM | 2,000 | 28 | 3.4×3.4×3.0 | Siemens Magnetom Trio Tim |
| Yale | 2,000 | 25 | 3.4×3.4×4.0 | Siemens Magnetom Trio Tim |

**Table S4** Sample sizes by site and diagnostic category. The number of participants from each scanning site is stratified by diagnostic group. Sites selected for the external testing set are indicated in parentheses

| Institution | ADHD Combined | ADHD Hyperactive/Impulsive | ADHD Inattentive | ASD | Neurotypical |
| --- | --- | --- | --- | --- | --- |
| Caltech | 0 | 0 | 0 | 12 | 11 |
| CMU | 0 | 0 | 0 | 1 | 1 |
| KKI | 14 | 0 | 5 | 9 | 83 |
| Leuven | 0 | 0 | 0 | 17 (testing) | 31 |
| Max_Mum | 0 | 0 | 0 | 5 | 13 |
| NYU | 94 | 1 (testing) | 49 | 66 | 202 |
| OHSU | 45 | 2 | 29 | 13 | 119 |
| Olin | 0 | 0 | 0 | 14 | 10 |
| Pitt | 0 | 0 | 0 | 17 | 92 |
| SBL | 0 | 0 | 0 | 1 | 1 |
| SDSU | 0 | 0 | 0 | 9 (testing) | 21 |
| Stanford | 0 | 0 | 0 | 15 | 14 |
| Trinity | 0 | 0 | 0 | 19 | 23 |
| UCLA | 0 | 0 | 0 | 34 | 39 |
| UM | 0 | 0 | 0 | 43 | 67 |
| USM | 0 | 0 | 0 | 29 | 27 |
| Yale | 0 | 0 | 0 | 19 | 23 |
| Peking | 2 (testing) | 0 | 12 (testing) | 0 | 99 |
| NeuroIMAGE | 7 (testing) | 6 | 1 | 0 | 23 |
| WashU | 0 | 0 | 0 | 0 | 20 (testing) |

**Table S5** Distribution of final image counts for each subgroup (age, gender) in training set, validation set and testing set, as well as the total dataset

| Dataset | Category | Total | ADHD Combined | ADHD  Hyperactive/Impulsive | ADHD Inattentive | ASD | Neurotypical |
| --- | --- | --- | --- | --- | --- | --- | --- |
| Total |  | 15,140 | 1,620 | 180 | 960 | 3,220 | 9,160 |
|  | **Age (years old)** | | | | | | |
|  | 0-8 | 2,470 | 600 | 10 | 160 | 260 | 1,440 |
|  | 9-12 | 6,190 | 800 | 120 | 670 | 940 | 3,660 |
|  | 13-17 | 4,320 | 190 | 20 | 120 | 1,280 | 2,710 |
|  | 18-26 | 2,160 | 30 | 30 | 10 | 740 | 1,350 |
|  | unknown | 0 | 0 | 0 | 0 | 0 | 0 |
|  | **Gender** | | | | | | |
|  | Female | 4,360 | 340 | 20 | 400 | 400 | 3,200 |
|  | Male | 10,760 | 1,260 | 160 | 560 | 2,820 | 5,960 |
|  | unknown | 20 | 20 | 0 | 0 | 0 | 0 |
| Training set |  | 11,496 | 1,224 | 64 | 672 | 2,368 | 7,168 |
|  | **Age (years old)** | | | | | | |
|  | 0-8 | 1,963 | 491 | 9 | 122 | 208 | 1,133 |
|  | 9-12 | 4,686 | 614 | 19 | 464 | 739 | 2,850 |
|  | 13-17 | 3,231 | 119 | 18 | 77 | 894 | 2,123 |
|  | 18-26 | 1,616 | 0 | 18 | 9 | 527 | 1,062 |
|  | unknown | 0 | 0 | 0 | 0 | 0 | 0 |
|  | **Gender** | | | | | | |
|  | Female | 3,417 | 259 | 18 | 270 | 307 | 2,563 |
|  | Male | 8,062 | 948 | 46 | 402 | 2,061 | 4,605 |
|  | unknown | 17 | 17 | 0 | 0 | 0 | 0 |
| Validation set |  | 2,874 | 306 | 16 | 168 | 592 | 1,792 |
|  | **Age (years old)** | | | | | | |
|  | 0-8 | 467 | 109 | 1 | 38 | 52 | 267 |
|  | 9-12 | 1,164 | 166 | 1 | 106 | 181 | 710 |
|  | 13-17 | 839 | 31 | 2 | 23 | 236 | 547 |
|  | 18-26 | 404 | 0 | 12 | 1 | 123 | 268 |
|  | unknown | 0 | 0 | 0 | 0 | 0 | 0 |
|  | **Gender** | | | | | | |
|  | Female | 803 | 71 | 2 | 70 | 73 | 587 |
|  | Male | 2,068 | 232 | 14 | 98 | 519 | 1,205 |
|  | unknown | 3 | 3 | 0 | 0 | 0 | 0 |
| Testing set |  | 770 | 90 | 100 | 120 | 260 | 200 |
|  | **Age (years old)** | | | | | | |
|  | 0-8 | 40 | 0 | 0 | 0 | 0 | 40 |
|  | 9-12 | 340 | 20 | 100 | 100 | 20 | 100 |
|  | 13-17 | 250 | 40 | 0 | 20 | 150 | 40 |
|  | 18-26 | 140 | 30 | 0 | 0 | 90 | 20 |
|  | unknown | 0 | 0 | 0 | 0 | 0 | 0 |
|  | **Gender** | | | | | | |
|  | Female | 140 | 10 | 0 | 60 | 20 | 50 |
|  | Male | 630 | 80 | 100 | 60 | 240 | 150 |
|  | unknown | 0 | 0 | 0 | 0 | 0 | 0 |

**Table S6** The input shape, newly added/modified layers and activation function incorporated into each pre-trained deep neural network and ADBrainNet

| Neural network | Input shape | Newly added/modified layers | Activation function |
| --- | --- | --- | --- |
| ADBrainNet | 64*64*3 | All | Softmax |
| AlexNet | 64*64*3 | Dense | Softmax |
| MobileNet | 224*224*3 | AveragePooling, Dense | Softmax |
| ResNet18 | 64*64*3 | Dense | Softmax |
| Xception | 128*128*3 | AveragePooling, Dense, Dropout | Softmax |

**Table S7** Details of class-specific data augmentation strategy

| Category | Augmentation Factor | Geometric Transformations | Color Enhancements | Intensity Level |
| --- | --- | --- | --- | --- |
| ADHD hyperactive/impulsive | 15* | Rotation (±30°, 50%) Horizontal flip (50%) Vertical flip (50%) | Brightness: 0.7-1.3  Contrast: 0.7-1.3  Sharpness: 0.7-1.3  Color balance: 0.8-1.2 | Strongest |
| ADHD combined | 4* | Horizontal flip (50%) | Brightness: 0.8-1.2 Contrast: 0.8-1.2 | Medium |
| ADHD inattentive | 3* | Horizontal flip (50%) | Brightness: 0.85-1.15 | Medium-Weak |
| ASD | 2* | Horizontal flip (30%) | Brightness: 0.9-1.1 | Light |
| Neurotypical | - | - | - | - |

**Table S8** The detailed performance of the baseline models without class imbalance mitigation on the external testing set. Metrics including accuracy, precision, recall, and F1-score are reported for each fold, along with the mean ± SD across the five-fold cross-validation

| Model | Accuracy (%) | Precision (%) | Recall (%) | F1 score (%) |
| --- | --- | --- | --- | --- |
| **ADBrainNet** | | | | |
| Fold1 | 33.51 | 38.93 | 33.51 | 23.61 |
| Fold2 | 25.32 | 40.36 | 25.32 | 11.45 |
| Fold3 | 25.84 | 40.45 | 25.84 | 11.12 |
| Fold4 | 23.90 | 6.84 | 23.90 | 10.27 |
| Fold5 | 32.21 | 40.95 | 32.21 | 21.95 |
| Average performance  (mean ± standard deviation) | 28.16±4.38 | 33.51±14.93 | 28.16±4.38 | 15.68±6.52 |
| **AlexNet** | | | | |
| Fold1 | 25.97 | 6.75 | 25.97 | 10.71 |
| Fold2 | 25.97 | 6.75 | 25.97 | 10.71 |
| Fold3 | 21.95 | 7.30 | 21.95 | 10.60 |
| Fold4 | 25.97 | 6.75 | 25.97 | 10.71 |
| Fold5 | 25.97 | 6.75 | 25.97 | 10.71 |
| Average performance  (mean ± standard deviation) | 25.17±1.80 | 6.86±0.25 | 25.17±1.80 | 10.69±0.05 |
| **MobileNet** | | | | |
| Fold1 | 25.97 | 6.75 | 25.97 | 10.71 |
| Fold2 | 25.97 | 6.75 | 25.97 | 10.71 |
| Fold3 | 31.82 | 34.98 | 31.82 | 23.64 |
| Fold4 | 25.97 | 6.75 | 25.97 | 10.71 |
| Fold5 | 25.58 | 6.67 | 25.58 | 10.58 |
| Average performance  (mean ± standard deviation) | 27.06±2.67 | 12.38±12.63 | 27.06±2.67 | 13.27±5.80 |
| **ResNet18** | | | | |
| Fold1 | 24.94 | 7.70 | 24.94 | 10.57 |
| Fold2 | 28.31 | 36.79 | 28.31 | 22.26 |
| Fold3 | 29.22 | 45.56 | 29.22 | 21.15 |
| Fold4 | 25.97 | 6.75 | 25.97 | 10.71 |
| Fold5 | 28.57 | 41.50 | 28.57 | 17.30 |
| Average performance  (mean ± standard deviation) | 27.40±1.84 | 27.66±18.91 | 27.40±1.84 | 16.40±5.57 |
| **Xception** | | | | |
| Fold1 | 25.97 | 67.47 | 25.97 | 10.71 |
| Fold2 | 25.97 | 67.47 | 25.97 | 10.71 |
| Fold3 | 25.97 | 67.47 | 25.97 | 10.71 |
| Fold4 | 25.97 | 67.47 | 25.97 | 10.71 |
| Fold5 | 25.97 | 67.47 | 25.97 | 10.71 |
| Average performance  (mean ± standard deviation) | 25.97±0.00 | 67.47±0.00 | 25.97±0.00 | 10.71±0.00 |

**Table S9** Per-class performance (precision, recall, and F1 score, in %) of the baseline models on the external testing set without class imbalance mitigation

|  | Model | ADHD combined | ADHD  hyperactive impulsive | ADHD inattentive | ASD | Neurotypical |
| --- | --- | --- | --- | --- | --- | --- |
| **ADBrainNet** | **Fold1** | | | | | |
|  | Precision | 0.00 | 0.00 | 0.00 | 93.55 | 28.25 |
|  | Recall | 0.00 | 0.00 | 0.00 | 22.31 | 100.00 |
|  | F1 score | 0.00 | 0.00 | 0.00 | 36.02 | 44.05 |
|  | **Fold2** | | | | | |
|  | Precision | 0.00 | 0.00 | 0.00 | 100.00 | 25.40 |
|  | Recall | 0.00 | 0.00 | 0.00 | 1.54 | 95.50 |
|  | F1 score | 0.00 | 0.00 | 0.00 | 3.03 | 40.13 |
|  | **Fold3** | | | | | |
|  | Precision | 0.00 | 0.00 | 0.00 | 100.00 | 25.75 |
|  | Recall | 0.00 | 0.00 | 0.00 | 0.77 | 98.50 |
|  | F1 score | 0.00 | 0.00 | 0.00 | 1.53 | 40.83 |
|  | **Fold4** | | | | | |
|  | Precision | 3.85 | 0.00 | 0.00 | 0.00 | 24.60 |
|  | Recall | 1.11 | 0.00 | 0.00 | 0.00 | 91.50 |
|  | F1 score | 1.72 | 0.00 | 0.00 | 0.00 | 38.77 |
|  | **Fold5** | | | | | |
|  | Precision | 0.00 | 0.00 | 0.00 | 100.00 | 27.64 |
|  | Recall | 0.00 | 0.00 | 0.00 | 18.85 | 99.50 |
|  | F1 score | 0.00 | 0.00 | 0.00 | 31.72 | 43.26 |
| **AlexNet** | **Fold1** | | | | | |
|  | Precision | 0.00 | 0.00 | 0.00 | 0.00 | 25.97 |
|  | Recall | 0.00 | 0.00 | 0.00 | 0.00 | 100.00 |
|  | F1 score | 0.00 | 0.00 | 0.00 | 0.00 | 41.24 |
|  | **Fold2** | | | | | |
|  | Precision | 0.00 | 0.00 | 0.00 | 0.00 | 25.97 |
|  | Recall | 0.00 | 0.00 | 0.00 | 0.00 | 11.00 |
|  | F1 score | 0.00 | 0.00 | 0.00 | 0.00 | 41.24 |
|  | **Fold3** | | | | | |
|  | Precision | 10.47 | 0.00 | 0.00 | 0.00 | 23.39 |
|  | Recall | 10.00 | 0.00 | 0.00 | 0.00 | 80.00 |
|  | F1 score | 10.23 | 0.00 | 0.00 | 0.00 | 36.20 |
|  | **Fold4** | | | | | |
|  | Precision | 0.00 | 0.00 | 0.00 | 0.00 | 25.97 |
|  | Recall | 0.00 | 0.00 | 0.00 | 0.00 | 100.00 |
|  | F1 score | 0.00 | 0.00 | 0.00 | 0.00 | 41.24 |
|  | **Fold5** | | | | | |
|  | Precision | 0.00 | 0.00 | 0.00 | 0.00 | 25.97 |
|  | Recall | 0.00 | 0.00 | 0.00 | 0.00 | 100.00 |
|  | F1 score | 0.00 | 0.00 | 0.00 | 0.00 | 41.24 |
| **MobileNet** | **Fold1** | | | | | |
|  | Precision | 0.00 | 0.00 | 0.00 | 0.00 | 25.97 |
|  | Recall | 0.00 | 0.00 | 0.00 | 0.00 | 100.00 |
|  | F1 score | 0.00 | 0.00 | 0.00 | 0.00 | 41.24 |
|  | **Fold2** | | | | | |
|  | Precision | 0.00 | 0.00 | 0.00 | 0.00 | 25.97 |
|  | Recall | 0.00 | 0.00 | 0.00 | 0.00 | 100.00 |
|  | F1 score | 0.00 | 0.00 | 0.00 | 0.00 | 41.24 |
|  | **Fold3** | | | | | |
|  | Precision | 0.00 | 0.00 | 16.67 | 75.00 | 27.18 |
|  | Recall | 0.00 | 0.00 | 3.33 | 23.08 | 90.50 |
|  | F1 score | 0.00 | 0.00 | 5.56 | 35.29 | 41.80 |
|  | **Fold4** | | | | | |
|  | Precision | 0.00 | 0.00 | 0.00 | 0.00 | 25.97 |
|  | Recall | 0.00 | 0.00 | 0.00 | 0.00 | 100.00 |
|  | F1 score | 0.00 | 0.00 | 0.00 | 0.00 | 41.24 |
|  | **Fold5** | | | | | |
|  | Precision | 0.00 | 0.00 | 0.00 | 0.00 | 25.68 |
|  | Recall | 0.00 | 0.00 | 0.00 | 0.00 | 98.50 |
|  | F1 score | 0.00 | 0.00 | 0.00 | 0.00 | 40.74 |
| **ResNet18** | **Fold1** | | | | | |
|  | Precision | 10.00 | 0.00 | 0.00 | 0.00 | 25.13 |
|  | Recall | 1.11 | 0.00 | 0.00 | 0.00 | 95.50 |
|  | F1 score | 2.00 | 0.00 | 0.00 | 0.00 | 39.79 |
|  | **Fold2** | | | | | |
|  | Precision | 16.00 | 0.00 | 31.71 | 69.84 | 24.61 |
|  | Recall | 4.44 | 0.00 | 10.83 | 16.92 | 78.50 |
|  | F1 score | 6.96 | 0.00 | 16.15 | 27.24 | 37.47 |
|  | **Fold3** | | | | | |
|  | Precision | 16.67 | 0.00 | 19.23 | 100.00 | 26.38 |
|  | Recall | 6.67 | 0.00 | 4.17 | 14.23 | 88.50 |
|  | F1 score | 9.52 | 0.00 | 6.85 | 24.92 | 40.64 |
|  | **Fold4** | | | | | |
|  | Precision | 0.00 | 0.00 | 0.00 | 0.00 | 25.97 |
|  | Recall | 0.00 | 0.00 | 0.00 | 0.00 | 100.00 |
|  | F1 score | 0.00 | 0.00 | 0.00 | 0.00 | 41.24 |
|  | **Fold5** | | | | | |
|  | Precision | 7.69 | 0.00 | 0.00 | 100.00 | 26.30 |
|  | Recall | 1.11 | 0.00 | 0.00 | 10.38 | 96.00 |
|  | F1 score | 1.94 | 0.00 | 0.00 | 18.82 | 41.29 |
| **Xception** | **Fold1** | | | | | |
|  | Precision | 0.00 | 0.00 | 0.00 | 0.00 | 25.97 |
|  | Recall | 0.00 | 0.00 | 0.00 | 0.00 | 100.00 |
|  | F1 score | 0.00 | 0.00 | 0.00 | 0.00 | 41.24 |
|  | **Fold2** | | | | | |
|  | Precision | 0.00 | 0.00 | 0.00 | 0.00 | 25.97 |
|  | Recall | 0.00 | 0.00 | 0.00 | 0.00 | 100.00 |
|  | F1 score | 0.00 | 0.00 | 0.00 | 0.00 | 41.24 |
|  | **Fold3** | | | | | |
|  | Precision | 0.00 | 0.00 | 0.00 | 0.00 | 25.97 |
|  | Recall | 0.00 | 0.00 | 0.00 | 0.00 | 100.00 |
|  | F1 score | 0.00 | 0.00 | 0.00 | 0.00 | 41.24 |
|  | **Fold4** | | | | | |
|  | Precision | 0.00 | 0.00 | 0.00 | 0.00 | 25.97 |
|  | Recall | 0.00 | 0.00 | 0.00 | 0.00 | 100.00 |
|  | F1 score | 0.00 | 0.00 | 0.00 | 0.00 | 41.24 |
|  | **Fold5** | | | | | |
|  | Precision | 0.00 | 0.00 | 0.00 | 0.00 | 25.97 |
|  | Recall | 0.00 | 0.00 | 0.00 | 0.00 | 100.00 |
|  | F1 score | 0.00 | 0.00 | 0.00 | 0.00 | 41.24 |

**Note:** All values represent performance metrics in percentage (%).

**Table S10** The detailed performance of the final models on the internal validation sets using class weighting and data augmentation to mitigate class imbalance. Metrics including accuracy, precision, recall, and F1 score are reported for each fold, along with the mean ± SD from five-fold cross-validation

| Model | Accuracy (%) | Precision (%) | Recall (%) | F1 score (%) |
| --- | --- | --- | --- | --- |
| **ADBrainNet** | | | | |
| Fold1 | 58.27 | 61.75 | 57.49 | 59.56 |
| Fold2 | 71.94 | 77.28 | 68.50 | 72.60 |
| Fold3 | 62.88 | 64.86 | 60.59 | 62.66 |
| Fold4 | 67.48 | 71.10 | 65.51 | 68.19 |
| Fold5 | 61.53 | 62.25 | 59.16 | 60.67 |
| Average performance  (mean ± standard deviation) | 64.42±4.78 | 67.45±5.94 | 62.25±4.11 | 64.74%±4.93 |
| **AlexNet** | | | | |
| Fold1 | 50.76 | 50.75 | 50.73 | 50.74 |
| Fold2 | 53.91 | 53.87 | 53.00 | 53.44 |
| Fold3 | 50.36 | 50.60 | 50.05 | 50.33 |
| Fold4 | 47.20 | 47.07 | 46.89 | 46.98 |
| Fold5 | 48.66 | 50.41 | 45.26 | 47.70 |
| Average performance  (mean ± standard deviation) | 50.18±2.26 | 50.54±2.15 | 49.19±2.77 | 49.84±2.31 |
| **MobileNet** | | | | |
| Fold1 | 45.23 | 45.36 | 44.43 | 44.90 |
| Fold2 | 52.57 | 52.77 | 52.37 | 52.58 |
| Fold3 | 54.66 | 55.23 | 53.31 | 54.26 |
| Fold4 | 48.92 | 49.12 | 47.63 | 48.37 |
| Fold5 | 50.28 | 51.39 | 45.65 | 48.36 |
| Average performance  (mean ± standard deviation) | 50.33±3.22 | 50.77±3.35 | 48.68±3.56 | 49.69±3.34 |
| **ResNet18** | | | | |
| Fold1 | 61.60 | 61.94 | 61.42 | 61.68 |
| Fold2 | 50.96 | 51.46 | 48.91 | 50.15 |
| Fold3 | 45.54 | 46.12 | 40.26 | 42.99 |
| Fold4 | 48.32 | 49.14 | 44.63 | 46.78 |
| Fold5 | 52.45 | 52.87 | 50.89 | 51.86 |
| Average performance  (mean ± standard deviation) | 51.77±0.06 | 52.31±0.06 | 49.22±0.08 | 50.69±0.07 |
| **Xception** | | | | |
| Fold1 | 54.41 | 55.05 | 46.06 | 50.16 |
| Fold2 | 49.75 | 51.22 | 39.83 | 44.80 |
| Fold3 | 50.34 | 52.09 | 45.43 | 48.51 |
| Fold4 | 50.10 | 51.87 | 42.00 | 46.42 |
| Fold5 | 56.08 | 58.18 | 53.93 | 55.97 |
| Average performance  (mean ± standard deviation) | 52.14±2.60 | 53.68±2.61 | 45.45±4.81 | 49.17%±3.86 |

**Table S11** Per-class performance (precision, recall, and F1 score, in %) of the final models on the external testing set after class imbalance mitigation

| Model | Fold number | ADHD combined | ADHD  hyperactive impulsive | ADHD inattentive | ASD | Neurotypical |
| --- | --- | --- | --- | --- | --- | --- |
| **ADBrainNet** | **Fold1** | | | | | |
|  | Precision | 39.60 | 70.00 | 34.21 | 100.00 | 44.27 |
|  | Recall | 4.44 | 70.00 | 32.50 | 77.69 | 56.00 |
|  | F1 score | 4.19 | 70.00 | 33.33 | 87.45 | 49.45 |
|  | **Fold2** | | | | | |
|  | Precision | 42.20 | 69.72 | 44.81 | 99.49 | 83.04 |
|  | Recall | 51.11 | 76.00 | 68.33 | 75.77 | 71.00 |
|  | F1 score | 46.23 | 72.73 | 54.13 | 86.03 | 76.55 |
|  | **Fold3** | | | | | |
|  | Precision | 23.53 | 78.02 | 26.86 | 99.18 | 43.11 |
|  | Recall | 8.89 | 71.00 | 39.17 | 93.46 | 48.50 |
|  | F1 score | 12.90 | 74.35 | 31.86 | 96.24 | 45.65 |
|  | **Fold4** | | | | | |
|  | Precision | 13.16 | 97.01 | 31.92 | 97.28 | 68.32 |
|  | Recall | 5.56 | 65.00 | 81.67 | 96.15 | 34.50 |
|  | F1 score | 7.81 | 77.84 | 45.90 | 96.71 | 45.85 |
|  | **Fold5** | | | | | |
|  | Precision | 17.74 | 71.30 | 29.63 | 100.00 | 42.86 |
|  | Recall | 12.22 | 82.00 | 33.33 | 87.31 | 49.50 |
|  | F1 score | 14.47 | 76.28 | 31.37 | 93.22 | 45.94 |
| **AlexNet** | **Fold1** | | | | | |
|  | Precision | 19.18 | 70.71 | 0.00 | 98.40 | 0.00 |
|  | Recall | 88.89 | 70.00 | 0.00 | 94.62 | 0.00 |
|  | F1 score | 31.56 | 70.35 | 0.00 | 96.47 | 0.00 |
|  | **Fold2** | | | | | |
|  | Precision | 12.11 | 94.64 | 30.82 | 95.51 | 6.25 |
|  | Recall | 34.44 | 53.00 | 40.83 | 98.08 | 10.00 |
|  | F1 score | 17.92 | 67.95 | 35.13 | 96.77 | 17.24 |
|  | **Fold3** | | | | | |
|  | Precision | 20.18 | 87.50 | 0.00 | 98.78 | 0.00 |
|  | Recall | 98.89 | 70.00 | 0.00 | 93.46 | 0.00 |
|  | F1 score | 33.52 | 77.78 | 0.00 | 96.05 | 0.00 |
|  | **Fold4** | | | | | |
|  | Precision | 18.13 | 0.00 | 0.00 | 94.81 | 0.00 |
|  | Recall | 98.89 | 0.00 | 0.00 | 98.46 | 0.00 |
|  | F1 score | 30.64 | 0.00 | 0.00 | 96.60 | 0.00 |
|  | **Fold5** | | | | | |
|  | Precision | 18.68 | 84.38 | 0.00 | 95.51 | 31.87 |
|  | Recall | 72.22 | 54.00 | 0.00 | 98.08 | 14.50 |
|  | F1 score | 29.68 | 65.85 | 0.00 | 96.77 | 19.93 |
| **MobileNet** | **Fold1** | | | | | |
|  | Precision | 19.43 | 100.00 | 17.65 | 100.00 | 16.43 |
|  | Recall | 67.78 | 75.00 | 2.50 | 30.00 | 23.50 |
|  | F1 score | 30.20 | 85.71 | 4.38 | 46.15 | 19.34 |
|  | **Fold2** | | | | | |
|  | Precision | 13.59 | 100.00 | 16.67 | 100.00 | 32.75 |
|  | Recall | 15.56 | 72.00 | 0.83 | 0.50 | 75.00 |
|  | F1 score | 14.51 | 83.72 | 1.59 | 67.00 | 45.59 |
|  | **Fold3** | | | | | |
|  | Precision | 17.86 | 70.25 | 25.00 | 99.11 | 33.81 |
|  | Recall | 5.56 | 85.00 | 5.00 | 42.69 | 82.00 |
|  | F1 score | 8.47 | 76.92 | 8.33 | 59.68 | 47.88 |
|  | **Fold4** | | | | | |
|  | Precision | 25.00 | 100.00 | 24.60 | 100.00 | 17.84 |
|  | Recall | 11.11 | 77.00 | 51.67 | 19.62 | 33.00 |
|  | F1 score | 2.13 | 87.01 | 33.33 | 32.80 | 22.53 |
|  | **Fold5** | | | | | |
|  | Precision | 23.08 | 57.82 | 22.43 | 100.00 | 29.38 |
|  | Recall | 20.00 | 85.00 | 20.00 | 6.15 | 62.00 |
|  | F1 score | 21.43 | 68.83 | 21.15 | 11.59 | 39.87 |
| **ResNet18** | **Fold1** | | | | | |
|  | Precision | 83.33 | 100.00 | 37.50 | 85.11 | 29.28 |
|  | Recall | 5.56 | 23.00 | 10.00 | 15.38 | 89.00 |
|  | F1 score | 6.67 | 37.40 | 15.79 | 26.06 | 44.06 |
|  | **Fold2** | | | | | |
|  | Precision | 17.30 | 94.12 | 21.43 | 99.27 | 29.02 |
|  | Recall | 35.56 | 16.00 | 2.50 | 52.31 | 60.50 |
|  | F1 score | 23.27 | 27.35 | 44.78 | 68.51 | 39.22 |
|  | **Fold3** | | | | | |
|  | Precision | 10.97 | 79.17 | 24.34 | 100.00 | 17.01 |
|  | Recall | 18.89 | 38.00 | 54.17 | 2.31 | 25.00 |
|  | F1 score | 13.88 | 51.35 | 33.59 | 4.51 | 20.24 |
|  | **Fold4** | | | | | |
|  | Precision | 10.32 | 75.86 | 28.92 | 96.71 | 28.77 |
|  | Recall | 14.44 | 44.00 | 20.00 | 56.54 | 50.50 |
|  | F1 score | 12.03 | 55.70 | 23.65 | 71.36 | 36.66 |
|  | **Fold5** | | | | | |
|  | Precision | 9.80 | 71.79 | 19.75 | 93.37 | 33.24 |
|  | Recall | 11.11 | 28.00 | 13.33 | 65.00 | 61.00 |
|  | F1 score | 10.42 | 40.29 | 15.92 | 76.64 | 43.03 |
| **Xception** | **Fold1** | | | | | |
|  | Precision | 13.10 | 95.52 | 31.43 | 94.84 | 26.60 |
|  | Recall | 36.67 | 64.00 | 9.17 | 77.69 | 27.00 |
|  | F1 score | 19.30 | 76.65 | 14.19 | 85.41 | 26.80 |
|  | **Fold2** | | | | | |
|  | Precision | 11.70 | 90.14 | 31.58 | 95.51 | 16.36 |
|  | Recall | 22.22 | 64.00 | 40.00 | 57.31 | 18.00 |
|  | F1 score | 15.33 | 74.85 | 35.29 | 71.63 | 17.14 |
|  | **Fold3** | | | | | |
|  | Precision | 19.12 | 98.46 | 14.29 | 96.34 | 30.53 |
|  | Recall | 53.33 | 64.00 | 3.33 | 60.77 | 40.00 |
|  | F1 score | 28.15 | 77.58 | 5.41 | 74.53 | 34.63 |
|  | **Fold4** | | | | | |
|  | Precision | 14.76 | 85.00 | 27.87 | 97.22 | 22.47 |
|  | Recall | 44.44 | 68.00 | 14.17 | 67.31 | 20.00 |
|  | F1 score | 22.16 | 75.56 | 18.78 | 79.55 | 21.16 |
|  | **Fold5** | | | | | |
|  | Precision | 17.94 | 96.83 | 17.24 | 94.58 | 31.75 |
|  | Recall | 44.44 | 61.00 | 4.17 | 73.85 | 40.00 |
|  | F1 score | 25.56 | 74.85 | 6.71 | 82.94 | 35.40 |

**Note:** All values represent performance metrics in percentage (%).

**Table S12** The detailed performance of ADBrainNet across different subgroups (age, gender) on the external testing set. Metrics including accuracy, precision, recall, and F1 score are reported for each fold, along with the mean ± SD from the five-fold cross-validation

| Category | Group (fold) | Accuray (%) | | Precision (%) | Recall (%) | | F1 score (%) |
| --- | --- | --- | --- | --- | --- | --- | --- |
| **Age (years old)** | **0-8** | | | | | | |
|  | Fold 1 | 52.27 | | 100.00 | 52.27 | | 68.66 |
|  | Fold 2 | 63.64 | | 100 | 63.64 | | 77.78 |
|  | Fold 3 | 31.82 | | 100 | 31.82 | | 48.28 |
|  | Fold 4 | 22.73 | | 100 | 22.73 | | 37.04 |
|  | Fold 5 | 45.45 | | 100 | 45.45 | | 62.5 |
|  | Average performance  (mean ± standard deviation) | 43.18±16.23 | | 100.00±0.00 | 43.18±16.23 | | 58.85±16.24 |
|  | **9-12** | | | | | | |
|  | Fold 1 | 50.30 | | 63.01 | 50.30 | | 54.36 |
|  | Fold 2 | 72.75 | | 79.14 | 72.75 | | 75.05 |
|  | Fold 3 | 55.09 | | 59.35 | 55.09 | | 56.48 |
|  | Fold 4 | 62.28 | | 69.88 | 62.28 | | 62.99 |
|  | Fold 5 | 55.39 | | 59.17 | 55.39 | | 56.67 |
|  | Average performance  (mean ± standard deviation) | 59.16±8.71 | | 66.11±8.48 | 59.16±8.71 | | 61.11±8.43 |
|  | **13-17** | | | | | | |
|  | Fold 1 | 62.65 | | 69.70 | 62.65 | | 64.76 |
|  | Fold 2 | 72.69 | | 85.46 | 72.69 | | 77.54 |
|  | Fold 3 | 69.88 | | 77.74 | 69.88 | | 70.68 |
|  | Fold 4 | 72.29 | | 78.18 | 72.29 | | 71.29 |
|  | Fold 5 | 67.47 | | 72.96 | 67.47 | | 69.56 |
|  | Average performance  (mean ± standard deviation) | 69.00±4.12 | | 76.81±5.98 | 69.00±4.12 | | 70.77±4.57 |
|  | **18-26** | | | | | | |
|  | Fold 1 | 55.94 | | 76.99 | 55.94 | | 61.29 |
|  | Fold 2 | 63.64 | | 90.72 | 63.64 | | 74.62 |
|  | Fold 3 | 65.73 | | 83.1 | 65.73 | | 67.5 |
|  | Fold 4 | 62.24 | | 84.57 | 62.24 | | 66.98 |
|  | Fold 5 | 60.14 | | 84.42 | 60.14 | | 64.65 |
|  | Average performance  (mean ± standard deviation) | 61.54±3.73 | | 84.00±4.89 | 61.54±3.73 | | 67.01±4.91 |
| **Gender** | **Female** | | | | | | |
|  | Fold 1 | 47.58 | 64.12 | | | 47.58 | 52.36 |
|  | Fold 2 | 71.21 | 77.61 | | | 71.21 | 73.11 |
|  | Fold 3 | 46.77 | 48.1 | | | 46.77 | 47.38 |
|  | Fold 4 | 62.9 | 66.11 | | | 62.9 | 62.16 |
|  | Fold 5 | 49.19 | 53.32 | | | 49.19 | 50.89 |
|  | Average performance  (mean ± standard deviation) | 55.53±10.96 | 61.85±11.55 | | | 55.53±10.96 | 57.18±10.46 |
|  | **Male** | | | | | | |
|  | Fold 1 | 56.97 | | 61.65 | 56.97 | | 58.43 |
|  | Fold 2 | 66.94 | | 82.91 | 66.94 | | 73.93 |
|  | Fold 3 | 63.16 | | 65.08 | 63.16 | | 63.15 |
|  | Fold 4 | 63.31 | | 71.63 | 63.31 | | 63.48 |
|  | Fold 5 | 61.61 | | 63.42 | 61.61 | | 61.92 |
|  | Average performance  (mean ± standard deviation) | 62.40±3.61 | | 68.94±8.67 | 62.40±3.61 | | 64.18±5.80 |

List of Abbreviations

| Abbreviation | Definition |
| --- | --- |
| Caltech | California Institute of Technology |
| CMU | Carnegie Mellon University |
| KKI | Kennedy Krieger Institute |
| Leuven | University of Leuven |
| Max_Mun | Ludwig Maximilians University Munich |
| NYU | NYU Langone Medical Center |
| OHSU | Oregon Health and Science University |
| Olin | Olin, Institute of Living at Hartford Hospital |
| Pitt/Pittsburgh | University of Pittsburgh School of Medicine |
| SBL | Social Brain Lab BCM MIC UMC Groningen and Netherlands Institute for Neurosciences |
| SDSU | San Diego State University |
| Stanford | Stanford University |
| Trinity | Trinity Centre for Health Sciences |
| UCLA | University of California, Los Angeles |
| UM | University of Michigan |
| USM | University of Utah School of Medicine |
| Yale | Yale Child Study Center |
| Peking | Peking university |
| NeuroIMAGE | NeuroIMAGE sample |
| WashU | Washington University in ST.Louis |
